# How Cognitive Impairment Affects Medication Management in Dental Settings

**DOI:** 10.1093/geroni/igaa057.2904

**Published:** 2020-12-16

**Authors:** Xi Chen, Jirakate Madiloggovit, Lisa Jacobson, Daniel Tranel, Carissa Comnick, Xianjin Xie

**Affiliations:** 1 University of Iowa, Iowa City, Iowa, United States; 2 University of Iowa Hospitals & Clinics, Iowa City, Iowa, United States; 3 University of Iowa Hospitals & Clinics, iowa City, Iowa, United States; 4 University of Iowa, Iowa city, Iowa, United States

## Abstract

Mishandling medications is commonly seen in persons with dementia, which can lead to poor treatment outcome and serious complications. Whether individuals with cognitive impairment can appropriately manage dental-related medication remains unknown, raising a liability concern for dentists who fail to recognize the patients at risk for mishandling their medications. To address this concern, we conducted a study with 51 participants with various cognitive impairment to describe their ability to handle dental-related medications. After cognitive assessment, participants were asked to set up an antibiotics pill-box and use a mouthwash as instructed, and their performance were scored. Number and type of prompts given to facilitate task completion were also documented. Mishandling of dental-related medications was common in participants with cognitive impairment. As expected, participants with poor cognition needed more assistance on handling their medications. Dentists should be aware of this concern and take it into consideration when treatment plan for these individuals. Part of a symposium sponsored by the Oral Health Interest Group.

